# Supplementary material for: Endophytic Trichoderma Species Isolated from Persea americana and Cinnamomum verum Roots Reduce Symptoms Caused by Phytophthora cinnamomi in Avocado
Source: Plants (Basel). 2020 Sep 17;9(9):1220. doi: 10.3390/plants9091220 (PMC7569818; doi:10.3390/plants9091220)
Supplement: Supplementary file 1 [file plants-09-01220-s001.pdf]

plants
MDPI

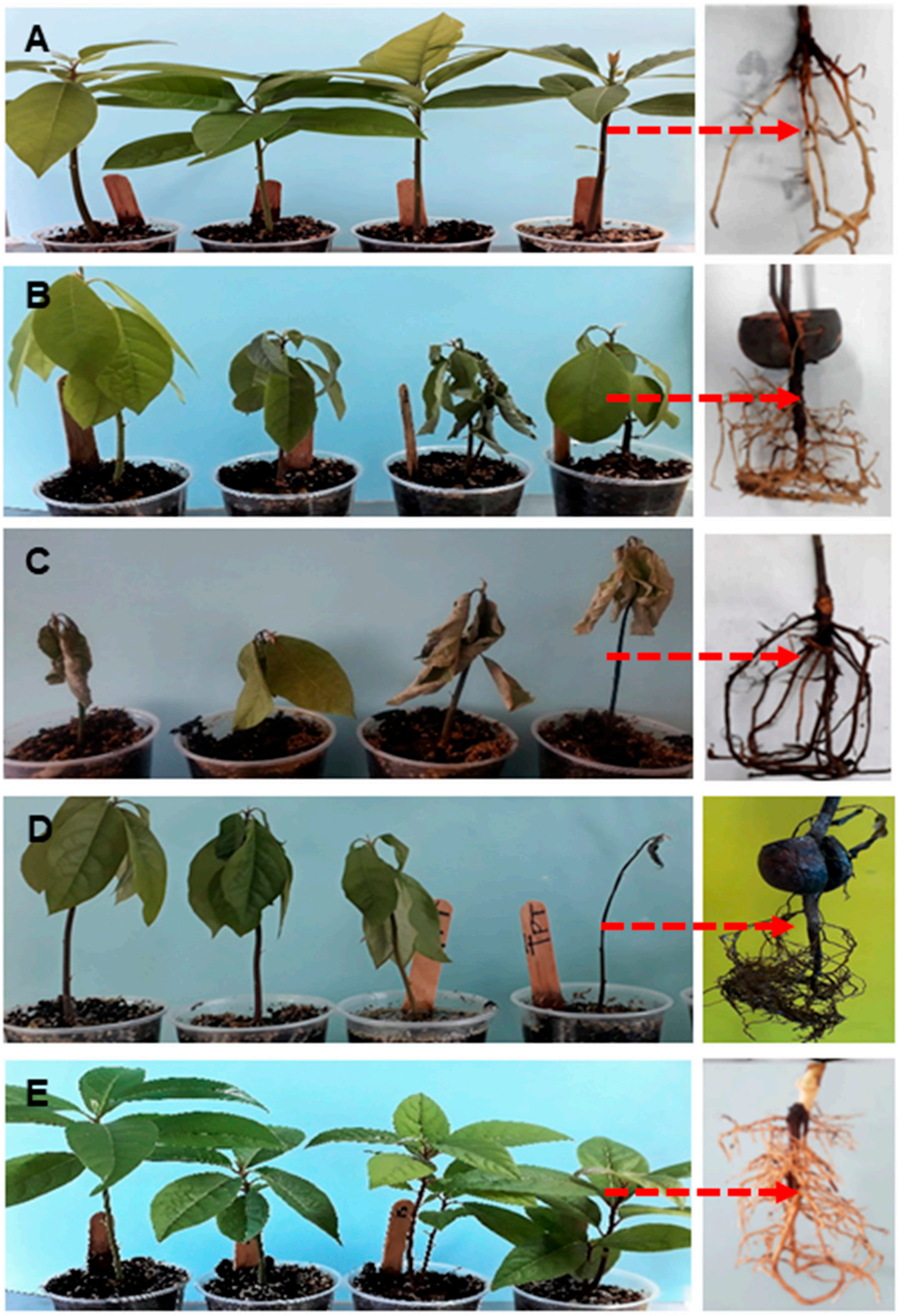

Figure S1. Symptoms of avocado seedlings inoculated with Trichoderma spp. and Phytophthora cinnamomi. (A) Asymptomatic seedlings inoculated individually with P. cinnamomi and Trichoderma asperellum, T. harzianum, T. hamatum, and T. koningiopsis. (B) Wilt symptoms observed in the combined P. cinnamomi and Trichoderma asperellum. (C) Dead seedlings. (D) Seedling inoculated with P. cinnamomi and without Trichoderma spp. (E) Control without pathogen. Red arrows indicate avocado root rot.

Table S1. Morphological characteristics of endophytic *Trichoderma* spp. strains isolated from avocado and cinnamon roots.

***Trichoderma* species**	**Colony Color**	**Colony Texture**	**Aerial Mycelium**	**Concentric Rings**	**Staining Colony Reverse**	**Conidial Shape**	**Conidial Size (µm)**	**Phialides Shape**	**Phialides Size (µm)**
T. asperellum T-AS1	Light to dark green	Dusty	Absence	3–4	Colorless	Obvoid to ellipsoidal	4.4 × 3.5	Slightly globose	2.3–2.8 × 10.2–11.8
T. asperellum T-AS2	Light to dark green	Dusty	Presence	3–4	Colorless	Obvoid to ellipsoidal	4.4 × 3.5	Slightly globose	2.3–2.8 × 10.2–11.8
T. asperellum T-AS6	Light to dark green	Dusty	Presence	3–4	Colorless	Obvoid to ellipsoidal	4.4 × 3.5	Slightly globose	2.3–2.8 × 10.2–11.8
T. asperellum T-AS7	Light to dark green	Dusty	Absence	3–4	Colorless	Obvoid to ellipsoidal	4.4 × 3.5	Slightly globose	2.3–2.8 × 10.2–11.8
T. hamatum T-A12	Dark green	Slightly adhered to the medium	Abundant	1–2	Faint yellow	Slightly ellipsoid	4.0 × 3.6	Slightly globose	2.3–2.9 × 10.1–11.9
T. harzianum T-H3	Dark green to grayish	Cottony	Abundant	Absence	Colorless	Subglobose	3.4–3.6 × 4.4–4.5	Globose	8–15 × 2–3
T. harzianum T-H4	Dark green to grayish	Cottony	Abundant	Absence	Colorless	Subglobose	3.4–3.6 × 4.4–4.5	Globose	8–15 × 2–3
T. harzianum T-H5	Dark green	Cottony	Abundant	Absence	Colorless	Subglobose	3.4–3.6 × 4.4–4.5	Globose	8–15 × 2–3
T. koningiopsis T-K8	Light to dark green	Slightly cottony and adhered to the medium	Presence	2–3	Colorless	Slightly subglobose	3.0–3.7 × 2.0–3.5	Slightly globose	3.0–4.5 × 2.7–3.5
T. koningiopsis T-K11	Light to dark green	Slightly cottony and adhered to the medium	Presence	2–3	Colorless	Slightly subglobose	3.0–3.5 × 2.0–3.5	Slightly globose	3.0–4.5 × 2.7–3.5
